# Is Mandibular Fossa Morphology and Articular Eminence Inclination Associated with Temporomandibular Dysfunction?

**Published:** 2016-06

**Authors:** Maryam Paknahad, Shoaleh Shahidi, Marzieh Akhlaghian, Masoud Abolvardi

**Affiliations:** 1 Prevention of Oral and Dental Disease Research Center, Dept. of Oral and Maxillofacial Radiology, School of Dentistry, Shiraz University of Medical Sciences, Shiraz, Iran.; 2 Dept. of Oral and Maxillofacial Radiology and Biomaterials Research Center, School of Dentistry, Shiraz University of Medical Sciences, Shiraz, Iran.; 3 Undergraduate Student, Dept. of Oral Radiology, School of Dentistry, Shiraz University of Medical Sciences, Shiraz, Iran.

**Keywords:** Cone Beam Computed, Tomography, Temporomandibular Joint, Temporomandibular Joint Disorder, Eminence

## Abstract

**Statement of the Problem:**

Finding a significant relationship between temporomandibular joint (TMJ) morphology and the incidence of temporomandibular dysfunction (TMD) may help early prediction and prevention of these problems.

**Purpose:**

The purpose of the present study was to determine the morphology of mandibular fossa and the articular eminence inclination in patients with TMD and in control group using cone beam computed tomography (CBCT).

**Materials and Method:**

The CBCT data of bilateral TMJs of 40 patients with TMD and 23 symptom-free cases were evaluated. The articular eminence inclination, as well as the glenoid fossa depth and width of the mandibular fossa were measured. The paired t-test was used to compare these values between two groups.

**Results:**

The articular eminence inclination and glenoid fossa width and depth were significantly higher in patients with TMD than in the control group (*p* < 0.05).

**Conclusion:**

The articular eminence inclination was steeper in patients with TMD than in the control group. Glenoid fossa width and depth were higher in patients with TMD than that in the control group. This information may shed light on the relationship between TMJ morphology and the incidence of TMD.

## Introduction

Temporomandibular joint dysfunction (TMD) is one of the most prevalent pathologies which may cause orofacial pain of non-dental origin.[1] The high prevalence of TMD makes it necessary to promote diagnostic and treatment methods.[2] TMD has different etiologies which are not yet known well.[3] Articular eminence morphology has been discussed as an etiologic factor for TMD in many studies.[4-8]

There are various diagnostic imaging techniques for the evaluation of temporomandibular joint (TMJ) structures. However, computerized tomography (CT) and cone beam computed tomography (CBCT) are the primary techniques of choice for optimal imaging of the osseous components. Although CT is widely used as a diagnostic tool in medicine, its application in dentistry is limited.[9] CBCT has a high dimensional accuracy in measuring maxillofacial structures including TMJ.[4, 7, 10-11] Therefore, CBCT was employed for the evaluation of TMJ morphology in the current study.

The relationship between the morphology of TMJ and TMD is a matter of controversy. Some studies have provided data suggesting that steeper articular eminence is a predisposing factor for TMD;[6, 10, 12-16]while other investigations failed to confirm this issue.[3, 17-22] Furthermore, there are some studies demonstrating that the healthy control group have steeper slope than patients with TMD.[2, 7, 23] To the best of our knowledge, there is not enough evidence showing the relationship between the glenoid fossa depth and width and the incidence of TMD. Finding a significant relationship between TMJ morphology and the incidence of TMD may help early prediction and prevention of these problems. Hence, this study aimed to investigate the relationship between the articular eminence inclination and also glenoid fossa width and depth according to gender in patients with TMD and symptom-free controls using CBCT.

## Materials and Method

This study was carried out in the Department of Oral and Maxillofacial Radiology at Shiraz Dental School, Iran. An expert radiologist examined the participants and divided them into two groups of symptomatic group and asymptomatic group based on Helkimo dysfunction index proposed by Helkimo in1974.[24] This index classifies the patients with signs and symptoms of TMJ disorders to measure and compare the severity of TMJ disorders in different populations and to assess the improvement of patients’ condition after treatment. This index is the functional evaluation of masticatory system which classifies individuals on the basis of 5 signs including pain during mandibular movement, TMJ function impairment, TMJ pain during palpation, impaired range of mandibular movement, and muscle tenderness.[24] (Table 1 and 22)

**Table 1:** Helkimo’s dysfunction index

**Clinical characteristics**			**Score**
1. Muscle tenderness No pain on palpation Tenderness to palpation in 1-3 palpation sites Tenderness to palpation in 4 or more sites			0 1 5
2. TMJ pain No tenderness to palpation Tenderness to palpation laterally Tenderness to palpation posteriorly			0 1 5
3. Pain during mandibular movement No pain Pain in 1 movement Pain in 2 or more movements			0 1 5
4. TMJ function impairment Smooth movement without sounds and deviation on opening or closing ≤2mm Sounds in one or both joints and/or deviation ≥2 mm on opening or closing Locking and/or luxation of the TMJ			0 1 5
5. Range of mandibular mobility A. Maximum opening of mouth >40 mm 30-39 mm <30 mm B. Maximum lateral movement to the right ≥7 mm 4-6 mm 0-3 mm C. Maximum lateral movement to the left ≥7 mm 4-6 mm 0-3 mm D. Maximum protrusion ≥7 mm 4-6 mm 0-3 mm	0 1 5 0 1 5 0 1 5 0 1 5	Sum A + B + C + D 0 points 1 1-4 points 5-20 points	0 1 5
Sum of 1+2+3+4+5			Di

**Table 2:** Grading of TMD based on Helkimo’s dysfunction index

**TMD** **degree**	**Sum of Helkimo’s** **Scores**	**Clinical degree of TMD**
Di o	0	No symptoms
Di I	1-4	Mild symptoms
Di II	5-9	Moderate symptoms
Di III	10-25	Acute/Serious symptoms

The asymptomatic group was composed of 23 participants (5 males and 18 females) without signs and symptoms related to TMD such as clicking, deviation during mouth opening with or without reduction, limited mouth opening, and tenderness of the lateral regions of the TMJ and masticatory muscles (masseter, temporal, medial pterygoid, and lateral pterygoid). In the control group, the patients who had any evidence of TMD in radiological examination were excluded from the present study. The symptomatic group consisted of 40 patients (12 males and 28 females) with signs and symptoms of TMD according to the Helkimo index for TMD. The exclusion criteria were the presence of congenital craniofacial abnormalities, fracture or pathology in the region of the articular eminence and/or any systemic diseases such as rheumatoid arthritis which may affect the joint morphology in both groups. In control and study group, patients with any prosthetic rehabilitation, history of orthodontic treatment, and also patients under 20 years old were excluded. The present study received approval from the Ethics Committee of Shiraz Dental School. A written consent was also obtained from each patient.

**Imaging procedures**

**Figure 1:**
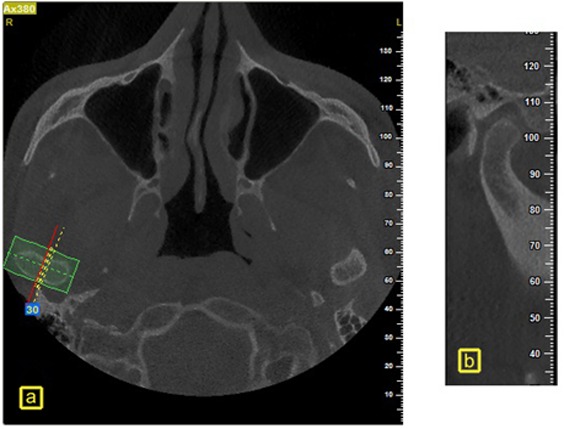
Reconstruction of CBCT sections in a sample case. a: Axial views in which the condylar process had its widest mediolateral diameter, b: Central sagittal section of the condyle

The CBCT images of the bilateral TMJ were taken by a NewTom VGi (QR s.r.l.; Verona, Italy) with a field of view 15×15cm. Images were obtained at 110 kVp, 3 mA and exposure time of 3.3 seconds. The patients were in standing position so that the Frankfort plane was held parallel to the horizontal plane on the lateral view. On the axial views, the section on which the condylar process had its widest mediolateral diameter was used for the secondary reconstruction of the sagittal slices. In this axial section, a line parallel to the long axis of the condylar process was drawn and the sagittal images were reconstructed at 0.5-mm slice interval and 0.5-mm thickness. The measurements were performed on the central sagittal section of the condyle (Figure 1).

**Measurements**

**Figure 2:**
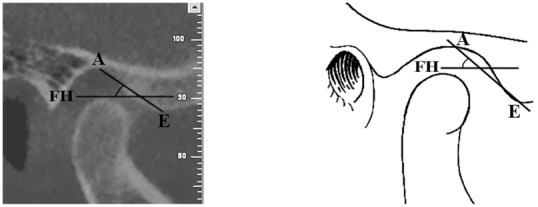
Measurement of articular eminence inclination with the top-roof line method in a sample case

In this study, the articular eminence inclination was measured by top-roof line method, i.e., the angle between Frankfort plane and the plane passing through the highest point in the roof of glenoid fossa and the lowest point at the crest of the articular eminence according to Shahidi *et al.* (Figure 2).[4]

**Figure 3:**
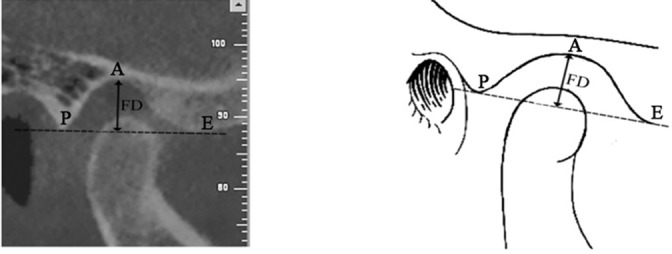
Measurement of fossa depth (FD) in a sample case, which is the perpendicular distance between the highest point of the fossa (A) and the line passing through the inferior point on the articular eminence (E) and the posterior part of glenoid process (P)

**Figure 4:**
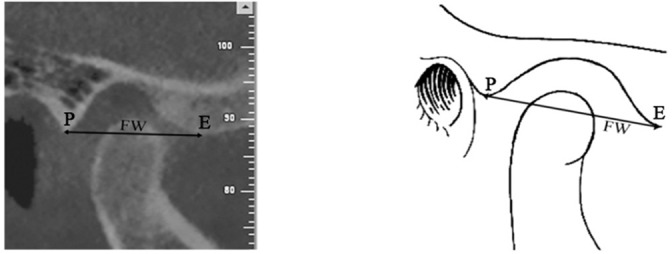
Measurement of fossa width (FW) in a sample case, which is the distance between the most inferior point on the articular eminence (E) and the posterior part of glenoid process (P)

The glenoid fossa depth was established by measuring the perpendicular distance between the highest point of fossa and the line passing through the most inferior point on the articular eminence and the posterior glenoid process (Figure 3). The glenoid fossa width was defined as the distance between the most inferior point on the articular eminence and the posterior glenoid process (Figure 4).

The same procedure and measurements were used on the condyle of both sides for each case.

**Measurement precision**

An expert maxillofacial radiologist measured the angles accurately by using NewTom NNT analysis software. To minimize the possible technical errors, all the measurements were repeated with one-week interval by the same observer. The observer was blinded to the allocation of patients to the TMD or symptom-free group.

**Statistical analyses**

The data analysis was conducted with the SPSS software version 17. The results of the first and second series of measurements were compared by paired t-test at a significance level of 0.05. The Student’s t-test was used to determine the possible statistically significant differences between the two groups according to gender. The Student’s t-test was used to determine the differences in the eminence inclination and the glenoid fossa depth and width between the patient and control groups according to their gender. P value≤ 0.05 was considered statistically significant. 

## Results

In the present study, patients' ages ranged from 25 to 50 years (mean 34.3 years) in the patient group and 21 to 57 years (42±2.93) in the control group. The results of the t-test showed no statistically significant difference between the two groups according to the chronological age (*p*= 0.126). Hence, the patients were matched for age in both groups. There was no statistically significant difference between the double measurements (*p*= 0.143). Therefore, the mean values were calculated for other statistical analysis. There was no statistically significant difference between the data of right and left joint of the groups (*p*= 0.167). Therefore, in each group, the data of both sides were joined together.

There were statistically significant differences between the patient and control groups in the articular eminence inclination (*p*= 0.001), and also the glenoid fossa depth (*p*= 0.008) and width (*p*< 0.001). All of these three values were higher in patients with TMD than in the control group (Table 3).

**Table 3:** The difference in articular eminence inclination, glenoid fossa depth and width values in patient and control groups

	**Patient group (N=80)**	**Control group (N=46)**	**p-value**
	Mean ± SD	Mean ± SD	
Articular eminence inclination (degree)	42.47 ± 7.91	37.33 ± 13.63	0.001*
Glenoid fossa depth (mm)	6.83 ± 1.68	6.07 ± 1.04	0.008*
Glenoid fossa width (mm)	18.8 ± 2.28	16.28 ± 2.70	<0.001*

N: number of joints, SD: standard deviation, mm: millimeter.

*: *p* < 0.05.

The eminence inclination values of males were lesser than those of females in both patient and control groups; however, these differences were not statistically significant (Table 4).

**Table 4:** Comparison of the eminence inclination and glenoid fossa depth and height values of the patient and control groups according to gender

		**Male**	**Female**	**p-value**
		**Mean ± SD**	**Mean ± SD**	
Control group N=46 (10 males and 36 females)	Articular eminence inclination (degree)	34.56 ± 6.21	38.10 ± 7.01	0.156
	Glenoid fossa depth (mm)	6.64 ± 0 .82	5.91 ± 1.05	0.047*
	Glenoid fossa width (mm)	15.63 ± 5.10	16.46 ± 1.61	0.625
Patient group N=80 (24 males and 56 females)	Articular eminence inclination (degree)	42.21 ± 9.49	42.59 ± 7.26	0.867
	Glenoid fossa depth (mm)	7.35 ± 1.326	6.60 ± 1.78	0.114
	Glenoid fossa width (mm)	19.17 ± 2.051	17.61 ± 2.23	0.014*

N: number of joints, SD: standard deviation, mm: millimeter.

* *P*< 0.05.

While males and females in patient group did not differ significantly in terms of the glenoid fossa depth values, there were statistically significant differences in the control group (*p*= 0.047), which showed higher values in males (Table 4).

No statistically significant difference was detected between the two genders in the glenoid fossa depth values in the control group; whereas, there was statistically significant difference in the patient group (*p*= 0.014) with males presenting higher values (Table 4).

## Discussion

Best-fit line method and top-roof line method are two different methods to measure the inclination of articular eminence both of which are reliable and have already been used in various studies.[4, 25] Kikuchi *et al.*,[25]used best-fit line method in which the eminence inclination was measured as the angle between the best-fit line on the posterior slope of articular eminence and the Frankfort horizontal plane. Shahidi *et al.*[4] used top-roof line method, which was the angle between the plane passing through the most superior point in the roof of fossa and the most inferior point at the crest of the articular eminence and Frankfort horizontal (FH) plane.[4] The authors suggested that this method focuses on the location of eminence crest relative to the fossa roof; while the best-fit line method focuses on the posterior surface of eminence. Therefore, the best-fit line method represents the actual condylar path; whereas, top-roof line depicts the morphology of articular eminence better. The present study used top-roof line method because its aim was to evaluate the TMJ morphology.

Concerning the high dose exposure and regarding the possibility of reconstruction of CBCT images and its ability to represent an image with improved quality in three orthogonal planes, selecting the best possible slice for our measurements on each view was imperative.[26] Most studies used the central sagittal slice of the condylar process because they demonstrated that this slice showed the steepest part of the articular eminence giving the best representation of the joint.[27-28] Thus, we chose the central sagittal slices of each condylar process in the measurements.

The inclination of articular eminence is described as the angle between the posterior wall of articular eminence and any horizontal plan.[29] It differs inter-individually and determines the condylar path during mandibular functions.[30] Association between the articular eminence inclination and many other factors such as age,[31] gender,[7, 27] TMD or internal derangement,[3, 8] malocclusion[32] and tooth loss[30] has been investigated in different studies. In the present study, we investigated the association between the articular eminence inclination and TMD. There is a controversy on the relationship between the inclination of articular eminence and TMD.

The articular eminence inclination was found to be steeper in the patient group than in control group. Many other studies also[6, 10, 12-14] demonstrated steeper eminence slope in the TMD group than in asymptomatic group. Meanwhile, few studies[7, 28] reported steeper eminence inclination in control group. A number of investigations[3, 17] found that the steepness of the articular eminence might not have a predisposing effect on the development of TMD.

Sülün *et al.*[6] proposed higher articular eminence as a predisposing factor for the development of disk displacement with reduction. Alkhader *et al.*[33] found higher articular eminence in TMJs with osseous abnormalities than those cases without abnormalities. Similarly, we found that fossa depth was higher in the TMD group than in control group. However, Ozkan *et al.*[3] proposed that eminence height might not be a predisposing factor for internal derangements of the joint. These controversies in articular eminence inclination and height could be due to differences in imaging techniques (CBCT versus MRI), methods of measurements, sample size, age range, and other differences between the populations.

Alkhader *et al.*[33] also proposed that fossa width was lower in TMJs with osseous abnormalities than the ones without such abnormalities. This is in contrast with our results showing higher fossa width in TMD group than in control group. This contrast could be attributed to the differences in case selection based on the inclusion and exclusion criteria. They only included patients with arthrogenic TMJ disorders; while, we included milder cases of TMD. We also set exclusion criteria such as congenital craniofacial abnormalities, fracture or pathology in TMJ[27] and any systemic diseases which might cause changes in bone morphology and consequently interfere with the results.[26] In line with some other studies,[27, 34-35]patients with any prosthetic rehabilitation or history of orthodontic treatment were not included in this study due to the probable etiological effect of these appliances on TMD. The articular eminence inclination completes its major growth by the age of 20.[36] Therefore, participants under 20 years of age were also excluded from this study.

Some studies reported a difference in articular eminence inclination or height according to gender.[7, 27, 30]Sümbüllü *et al.*[7] and Ilguy *et al.*[27] found that eminence inclination and height values of males were higher than those of females. Similarly, we found that eminence height (glenoid fossa depth) values of males were higher than those of females which was significant in the control group, but not in the patient group. In contrast with previous studies, this study found higher eminence inclination in females in both patient and control groups. These results confirm the sexual dimorphism which may be related to the variation of the amount of masticatory force affecting the joint according to gender.[7] Controversial results reported that the effect of gender in different studies could be due to the improper gender distribution of the patients. In our study as well as many others,[7, 27] the number of females was higher than males and this may be the underlying cause of the prevalence of TMD among females, as already reported in some studies.[7]

This study was subjected to some limitations such as improper distribution of the patients by gender and age. There were more females than males because of the higher incidence of TMD in females.[37] It was not certainly confirmed that the morphology is the cause or effect of TMD. Undoubtedly, additional studies with larger sample size are required to evaluate this relation and to resolve the mentioned controversy about eminence inclination. Further examinations are also necessary to be performed over a broader range of ages to find the effect of aging on TMJ; particularly the morphology of its components and the possible consequences such as TMD.

## Conclusion

In conclusion, inclination of articular eminence was steeper in patients with TMD. These patients also showed higher values of glenoid fossa depth and width. This information may shed light on the relationship between TMJ morphology and the incidence of TMD. 
